# Ammonium Gemini Surfactants Form Complexes with Model Oligomers of siRNA and dsDNA

**DOI:** 10.3390/ijms20225546

**Published:** 2019-11-07

**Authors:** Weronika Andrzejewska, Michalina Wilkowska, Andrzej Skrzypczak, Maciej Kozak

**Affiliations:** 1 Department of Macromolecular Physics, Faculty of Physics, Adam Mickiewicz University, Uniwersytetu Poznańskiego 2, 61-614 Poznań, Poland; weronika.andrzejewska@amu.edu.pl (W.A.); ms65293@amu.edu.pl (M.W.); 2 NanoBioMedical Centre, Adam Mickiewicz University, Wszechnicy Piastowskiej 3, 61-614 Poznań, Poland; 3 Faculty of Chemical Technology, Poznan University of Technology, Berdychowo 4, 60-965 Poznań, Poland; andrzej.skrzypczak@put.poznan.pl; 4 Joint SAXS Laboratory, Adam Mickiewicz University, Uniwersytetu Poznańskiego 2, 61-614 Poznań, Poland

**Keywords:** gene therapy, siRNA, DNA, lipoplexes

## Abstract

Dimeric cationic surfactants (gemini-type) are a group of amphiphilic compounds with potential use in gene therapy as effective carriers for nucleic acid transfection (i.e., siRNA, DNA, and plasmid DNA). Our studies have shown the formation of lipoplexes composed of alkanediyl-α,ω-bis[(oxymethyl)dimethyldodecylammonium] chlorides and selected 21-base-pair nucleic acid (dsDNA and siRNA) oligomers. To examine the structure and physicochemical properties of these systems, optical microscopy, circular dichroism spectroscopy (CD), small-angle X-ray scattering of synchrotron radiation (SR-SAXS), and agarose gel electrophoresis (AGE) were used. The lengths of spacer groups of the studied surfactants had a significant influence on the surfactants’ complexing properties. The lowest charge ratio (p/n) at which stable lipoplexes were observed was 1.5 and the most frequently occurring microstructure of these lipoplexes were cubic and micellar phases for dsDNA and siRNA, respectively. The cytotoxicity tests on HeLa cells indicated the non-toxic concentration of surfactants to be at approximately 10 µM. The dicationic gemini surfactants studied form complexes with siRNA and dsDNA oligomers; however, the complexation process is more effective towards siRNA. Therefore these systems could be applied as transfection systems for therapeutic nucleic acids.

## 1. Introduction

Gene therapy offers many methods for insertion of DNA, RNA fragments, or chimeric DNA–RNA systems into target cells [1,2]. The general idea is to apply short synthetic fragments of nucleic acids (transgenes) like siRNA, that, through RNA interference, would specifically exclude the expression of selected genes [3,4,5,6,7]. Unfortunately, the methods used to transfer these short oligomers into cells are still not satisfactory. Immune response mechanisms that cause problems in safe and effective insertion of transgenes within the pathological cells have had impeding effect [2,7]. In view of the above, the main purpose of the research is to devise suitable transfection carriers that could safely and easily bypass all natural cellular barriers. The use of delivery systems based on lipids or surfactants is very promising [8]. A group of chemical compounds showing the required properties are dimeric (gemini) surfactants [9,10,11,12]. Dicationic surfactant molecules in appropriate conditions (aqueous solution, pH, and ionic strength), and in the presence of nucleic acids, can form lipoplexes organized in supramolecular microstructures like hexagonal, lamellar, or cubic phases [13,14,15,16,17]. 

The influence of molecular architecture of dimeric surfactants on their ability to form stable complexes with nucleic acids is intensively studied, both in terms of the spacer group width, the length of alkyl chains, or the type of polar group. A wide spectrum of modulation possibilities of the architecture of gemini surfactant molecules allows for the selection of the right structure for the appropriate ability to bind nucleic acids [18,19,20,21,22,23,24,25]. Therefore, the aim of our research was to determine the complexation conditions and the structure of complexes formed by alkanediyl-α,ω-bis[(oxymethyl)dimethyldodecylammonium] chlorides and selected 21-base-pair nucleic acid (dsDNA and siRNA) oligomers.

## 2. Results

**Figure 1:**
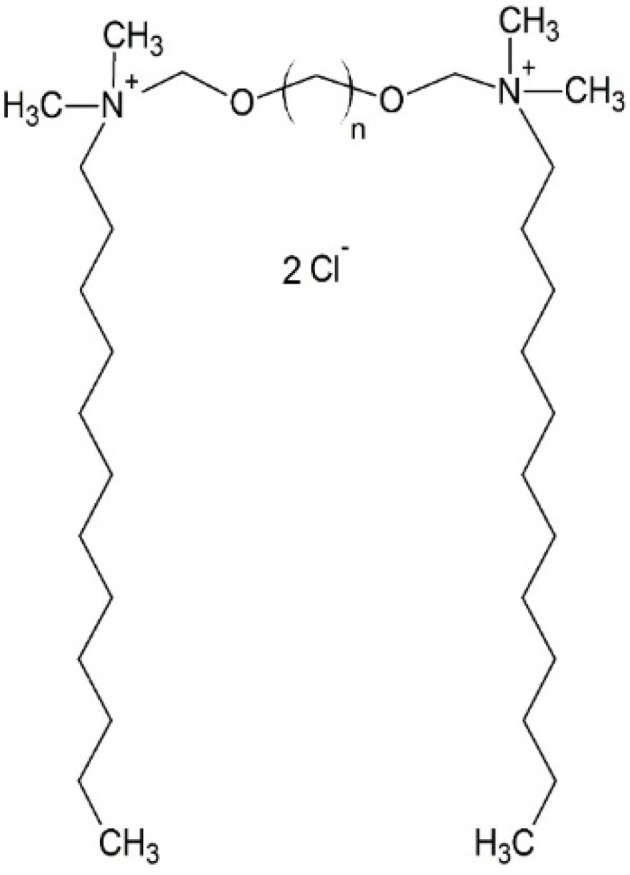
Chemical structure of alkanediyl-α,ω-bis[(oxymethyl)dimethyldodecylammonium] chlorides (C12NCn, *n* = 2–12).

For these studies, ten alkanediyl-α,ω-bis[(oxymethyl)dimethyldodecylammonium] chlorides (denoted as: C12NCn, where *n* is the number of methylene groups in the alkane spacer; *n* = 2, 3, 4, 5, 6, 7, 8, 9, 10, and 12) were selected (Figure 1) [26,27]. A single molecule of the studied amphiphilic compounds contain two head groups, with positively charged nitrogen atoms connected with two identical dodecyl side chains (hydrophobic tails), separated by the alkane spacer group of variable length. 

Oligomers of the nucleic acids used for this study were synthesized by Future Synthesis (Poznan, Poland). The siRNA 21-base-pair oligomer contains the following sequence: 

5′GUUACGACAAUCCUGUUUCdTdT3′ (sense) 

3’dTdTCAAUGCUGUUAGGACAAAG5’ (antisense). 

The sequence was chosen because it takes part in the silencing of the specific DMPK gene region [28]. We also tested the complementary dsDNA: 

5′GTTACGACAATCCTGTTTCTT3′ (sense)

3′TTCAATGCTGTTAGGACAAAG5′ (antisense). 

These oligomers have been previously successfully used by us for tests of imidazolium gemini surfactants as components of nucleic acids delivery systems [29,30].

The general complexation procedure was identical to that used for previously tested imidazolium gemini surfactants [29]. All compounds were dissolved in a 10 mM phosphate buffer (pH 7) for all experiments. To obtain desirable systems, mixtures of stock solutions of siRNA and dsDNA (concentration 98 µM) and surfactants in appropriate concentrations were prepared. The volume ratio of the components was 1:1. The samples were characterized by the p/n parameter (1) [31,32], defined as the positive charge at the surfactant head groups (p) and the negative charge accumulated on the surface of the sugar–phosphate backbone of the studied oligomers (n):

p/n = (c_surf_ ∙ n_+_) / (c_oligo_ ∙ n_−_), (1)

where n+ and n− are the numbers of positive and negative charges, c_surf_ and c_oligo_ are the concentrations of the surfactant and siRNA or dsDNA, respectively. The complexation time was approximately 25 min. Before the preparation of lipoplexes, all solutions of dicationic surfactants were sonicated for 15 min to homogenize the solutions.

### 2.1. Obtained Lipoplexes

**Figure 2:**
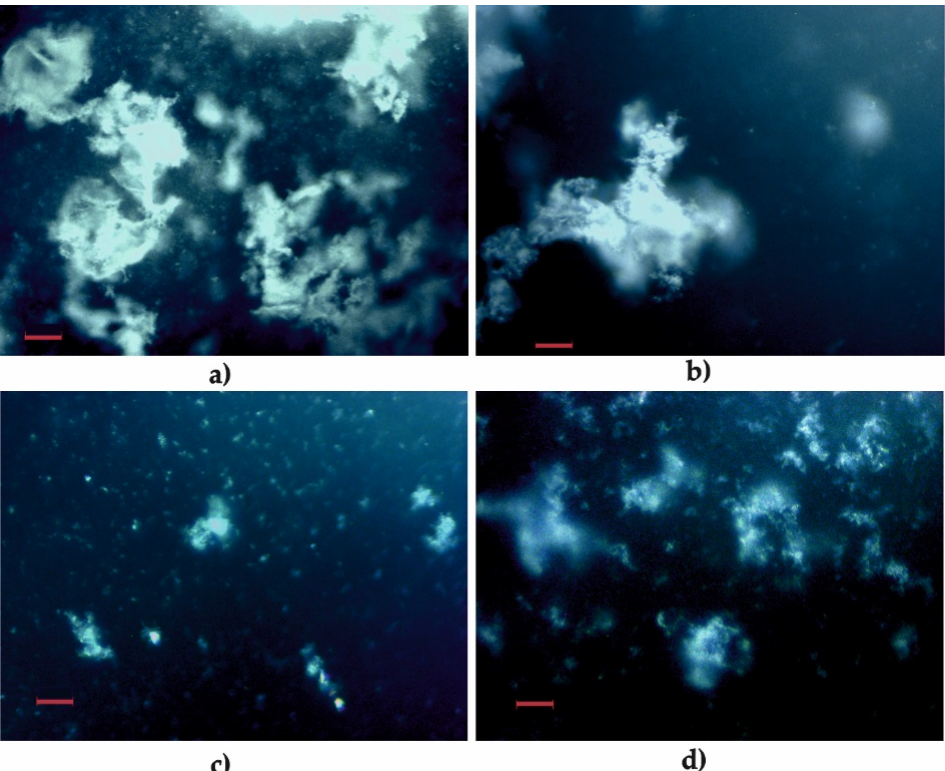
Optical microscope images of several tested mixtures: (**a**) C12NC2/dsDNA; (**b**) C12NC7/dsDNA; (**c**) C12NC9/dsDNA; and (**d**) C12NC12/dsDNA in p/n = 4. Magnification was 200×. Scale bars represent 50 µm.

The mixtures of siRNA and dsDNA oligomers with C12NCn gemini surfactants were first analyzed using optical microscopy (OM). In the highest concentrations of the amphiphile (p/n = 2–4), the tested mixtures were not transparent due to the formation of spatial structures visible even on a macroscopic scale (see Figure 2). This effect is a result of the self-organization process of the siRNA or dsDNA polyanions, and the positively charged surfactants within the lipoplexes formed in the solution. 

### 2.2. Binding Capacity of C12NCn with siRNA and dsDNA

**Figure 3:**
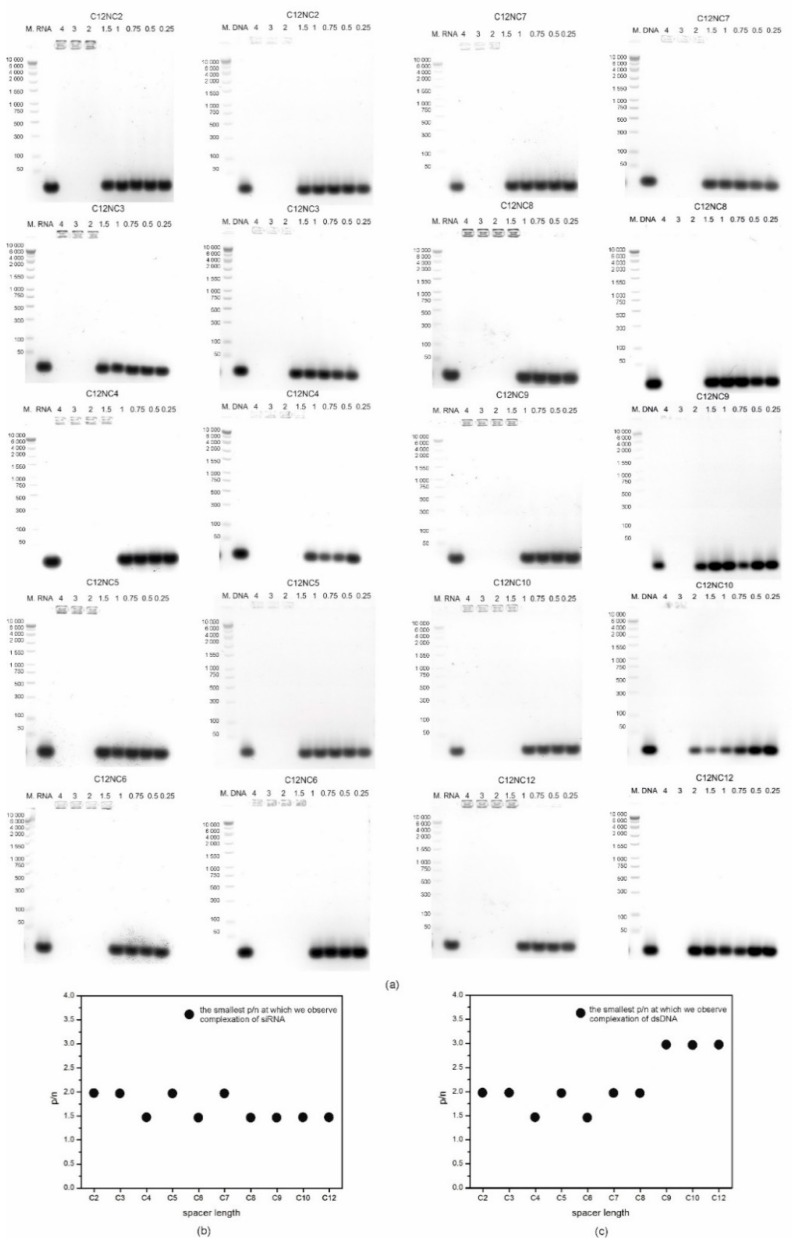
Agarose gel electrophoresis of C12NCn with siRNA and dsDNA (**a**). Comparison of gel electrophoresis results for all tested surfactants with siRNA (**b**) and dsDNA (**c**). M is a molecular weight marker.

The mixtures of surfactants with selected siRNA or dsDNA oligomers, prepared as above described, were separated in the agarose gel, and the final effects of electrophoretic separation are shown in Figure 3a. Nucleic acid oligomers in the native form are polyanions; therefore, they should move towards the positive pole in the electrophoretic gel [33,34]. The complexation efficiencies of siRNA and dsDNA oligomers by the tested C12NCn surfactants, studied in the gel electrophoresis experiment, were compared in order to identify a system with the lowest p/n values (Figure 3b,c). It was shown that the length of the surfactant spacer group significantly influences the nucleic acids binding properties by amphiphilic molecules. For the group of amphiphilic molecules with short spacers (C12NC2-C12NC7), there was no significant difference in binding of siRNA and dsDNA oligomers, which complexed usually in the p/n range 1.5–2. For the surfactants with longer spacer groups (i.e., the ones from C12NC8 to C12NC12), we observed a small decrease in the binding efficiency of dsDNA relative to that of siRNA, which was visible at p/n = 3. Comparison of the gel electrophoresis results also showed that C12NCn surfactants with an even number (*n*) of methylene segments in the spacer group exhibited better complexing properties.

### 2.3. Conformational Changes in Oligonucleotides Induced by Surfactants

**Figure 4:**
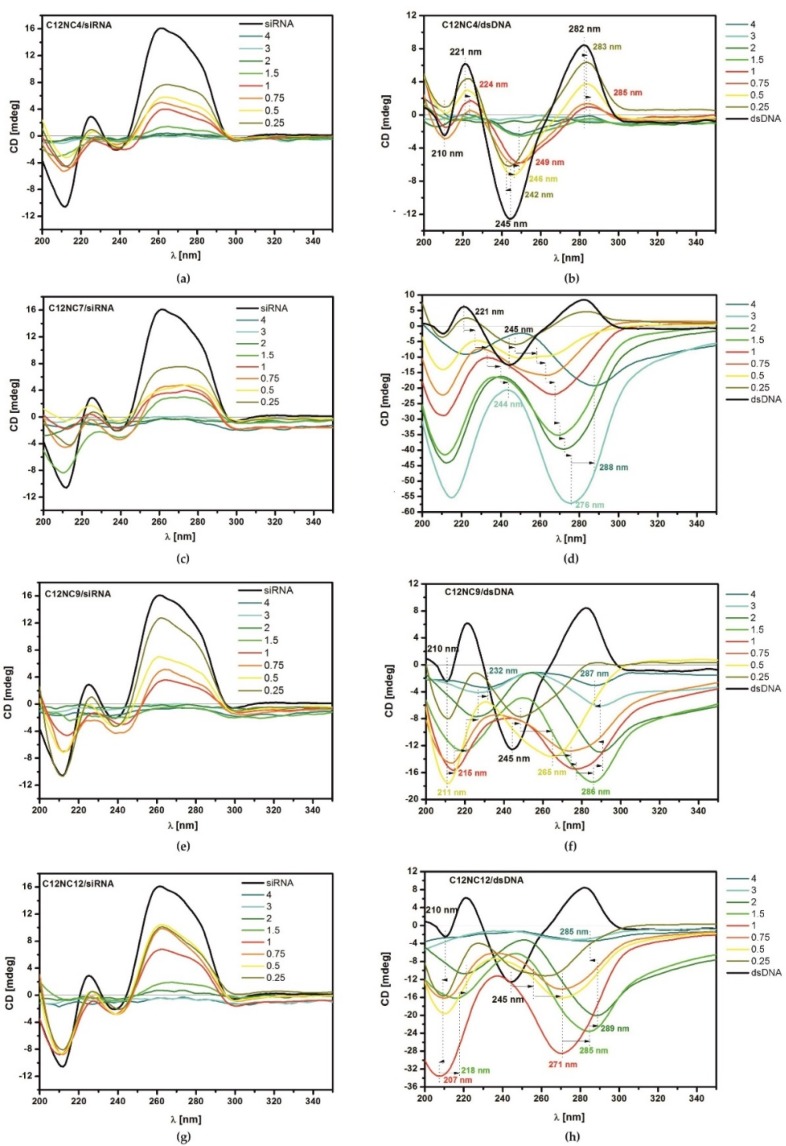
Circular dichroism results obtained for selected tested mixtures of C12NCn with siRNA (**a**,**c**,**e**,**g**) and dsDNA (**b**,**d**,**f**,**h**). The strongest signal which can be assigned to the Ψ-DNA phase was observed for C12NC7 for p/n = 3 (**d**).

The results of circular dichroism (CD) spectroscopy revealed conformational changes in the studied dsDNA and siRNA oligomers. Circular dichroism spectra obtained for the mixtures of siRNA or dsDNA with C12NCn showed significant changes in the conformation of oligomers resulting from the interaction with the tested amphiphilic molecules. For the native fully-hydrated siRNA, the recorded spectrum indicates the conformation of the dextrorotatory A-RNA helix, as the maximum of the CD signal was observed at 260 nm and the minimum at 211 nm. Depending on the C12NCn surfactant concentration (expressed in p/n ratio), the deformation of the CD spectra showed a different character (Figure 4a–d). Generally, the addition of a small amount of surfactant (p/n = 0.25–1.5 or 2) resulted in decreased intensity of the main bands, both negative and positive. For higher concentrations of C12NCn (p/n = 3–4), we observed an almost complete disappearance of all bands in the CD spectrum.

The length of the spacer group between polar head groups of the surfactant also influenced the shape of the CD spectra, recorded for the tested mixtures. The addition of some surfactants (C12NC2, C12NC3, C12NC5, and C12NC7) to the siRNA oligomer solution resulted in equalization of intensities of the two components of the positive CD band at 260 and 280 nm (Figure 4c), and flattening of the curve at p/n = 2, which was not observed for other surfactants.

The fully-hydrated dsDNA molecule was characterized by a conserved CD spectrum, with a symmetrical band at 260 nm, which is characteristic of the B-DNA form. Maximum amplitudes of the CD signal occurred at around 282 and 221 nm, while the minima at 245 and 210 nm [35,36,37]. The conformation of the dsDNA oligomer in the mixtures with the gemini surfactants tested differed depending on the length of the spacer group. The surfactants with shorter spacer groups (C12NC2–C12NC6) exhibited a similar effect on the conformation of dsDNA oligomer. For these surfactants we observed a gradual disappearance of individual bands in the CD spectrum and a series of small shifts of maxima and minima with increasing concentration of the surfactant, even up to complete loss of the CD signal (Figure 4a). For the remaining tested surfactants (C12NC7–C12NC12), significant shifts of individual bands in the CD spectra in the wavelength range from 210 to 218 nm (first minimum), from 221 to 245 nm (first maximum), and from 245 to 290 nm (second minimum) were noted, and the second maximum at 282 nm disappeared (Figure 4f–h). For the group of C12NCn surfactants, we did not observe a complete loss of the CD signal, even for the highest p/n ratios. However, the reversal of the entire CD spectrum took place. Of particular interest is the CD spectrum recorded for the dsDNA oligomer lipoplex with C12NC7 surfactant (Figure 4d). It was characterized by the signal intensity much higher than those noted for the other studied systems. With increasing concentration of the surfactant, the characteristic extrema in CD curves gradually were shifted towards longer wavelengths (at p/n = 4, the shift was 43 nm). This shift was accompanied by a very large, negative amplification of the characteristic bands at 210 and 245 nm, with a total disappearance of the positive band around 282 nm. The reduction of the CD signal intensity in the entire spectrum for the lipoplex formed at p/n = 4 was caused probably by the increase in turbidity of the solution [35].

### 2.4. Structures of C12NCn/siRNA and C12NCn/dsDNA Systems

**Figure 5:**
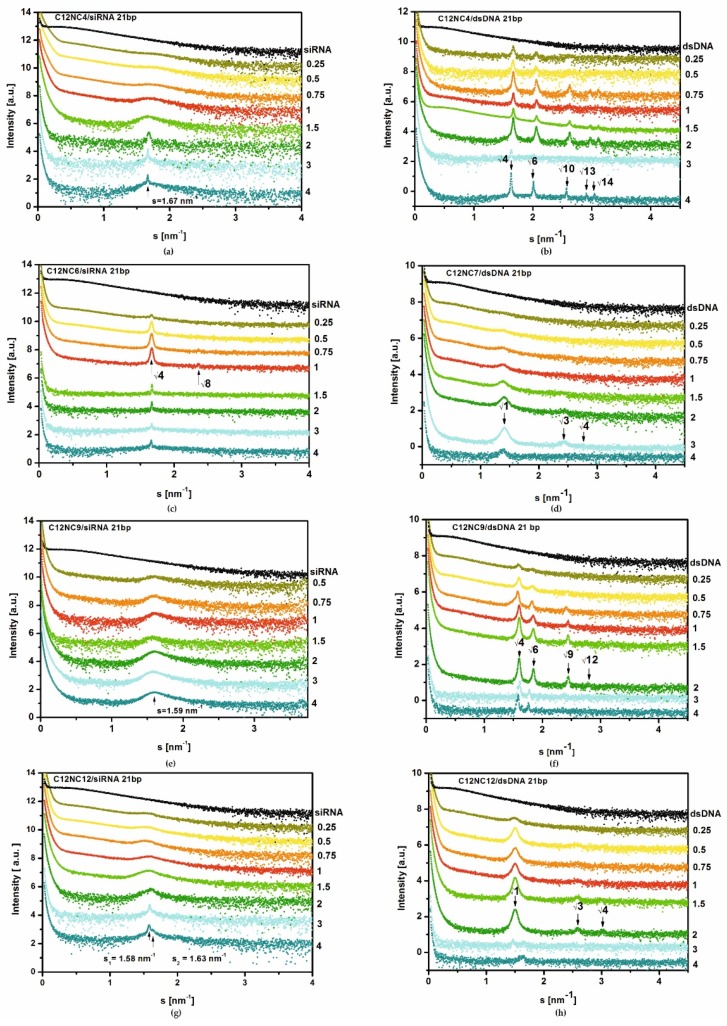
SR-SAXS curves recorded for selected mixtures of C12NCn with siRNA (**a**,**c**,**e**,**g**) and dsDNA (**b**,**d**,**f**,**h**) oligomers.

Structural parameters of lipoplexes siRNA and dsDNA with C12NCn surfactants were analyzed using the method of small-angle X-ray scattering of synchrotron radiation (SR-SAXS). The SAXS curves recorded for studied C12NCn lipoplexes are shown in Figure 5. The coexistence of amphiphilic molecules and negatively charged siRNA or dsDNA oligomers in solution resulted in self-assembly of both components in the entire volume of solutions. The SAXS curves collected for the tested siRNA/C12NCn lipoplexes showed mostly the presence of a micellar phase (Figure 5e). The appropriate scattering maximum in SAXS curve oscillated in the range 1.59 ≤ s ≥ 1.69 nm^−1^. The peak width observed in SAXS curves recorded for individual mixtures indicated a size distribution of the formed micelles. One sharp scattering peak, clearly visible on the SAXS curves (Figure 5a), was recorded for all lipoplexes of siRNAs with C12NC4 (s = 1.67 nm^−1^, d = 3.76 nm). This effect may indicate development of the lamellar phase in this system. Additionally, for siRNAs lipoplexes obtained with C12NC3, C12NC6, C12NC7, and C12NC12, the SAXS curves showed diffraction peaks. For the siRNA/C12NC3 system these peaks were visible at s_1_ = 1.62 nm^−1^ and s_2_ = 2.30 nm^−1^ (d_200_ = 3.88 nm and d_220_ = 2.73 nm). The ratio of peak positions was √4:√8, which suggests the presence of a cubic phase in the solution. The unit cell parameter of this phase was a = 7.76 nm. The identical ratio of the diffraction peak positions was also obtained for the SAXS curve for the siRNAs/C12NC6 system (peak positions: s_1_ = 1.67 nm^−1^ and s_2_ = 2.36 nm^−1^; d_200_ = 3.76 nm and d_220_ = 2.66 nm) (Figure 5c). The unit cell parameter a, characterizing this cubic phase, was 7.52 nm. For siRNA/C12NC7 lipoplexes, the diffraction peaks were noted at s_1_ = 1.48 nm^−1^ and s_2_ = 1.64 nm^−1^ (d_1_ = 4.25 nm and d_2_ = 3.83 nm), while for siRNA/C12NC12 system, the diffraction peaks appeared at s_1_ = 1.58 nm^−1^ and s_2_ = 1.63 nm^−1^ (d_1_ = 3.98 nm and d_2_ = 3.85 nm) (Figure 5g). For both C12NC7 and C12NC12 systems, the unambiguous assignment of the phase, based on the position of diffraction peaks, was not possible (Figure 5d,h).

The results of SAXS studies for mixtures of C12NCn surfactants with dsDNA oligomer (Figure 5) were different than for siRNA lipoplexes. For lipoplexes based on the surfactants containing the shortest spacer groups (C12NC2-C12NC4), the SAXS experiment showed the presence of a cubic phase, most likely Pm3n (Q^223^) (Figure 5b) [15,17,38]. The unit cell parameter determined for this phase was in the range a = 7.71–7.76 nm.

For dsDNA mixtures with C12NC5–C12NC6 and C12NC8–C12NC10 surfactants, on the basis of positions of observed diffraction peaks (Table 1, Figure 5), another cubic phase Pn3m (Q^224^) was identified (Figure 5f) [15,17,39,40]. The unit cell parameter characterizing this phase was in the range of a = 7.71–7.90 nm. For the other two dsDNA lipoplexes (with C12NC7 or C12NC12), on the basis of the scattering data, we succeeded in assigning the hexagonal phase with the parameters a = 5.15 nm, (C12NC7) and a = 4.84 nm (C12NC12) [11,39,41,42]. 

### 2.5. Toxicity of C12NCn Towards HeLa Cells

**Figure 6:**
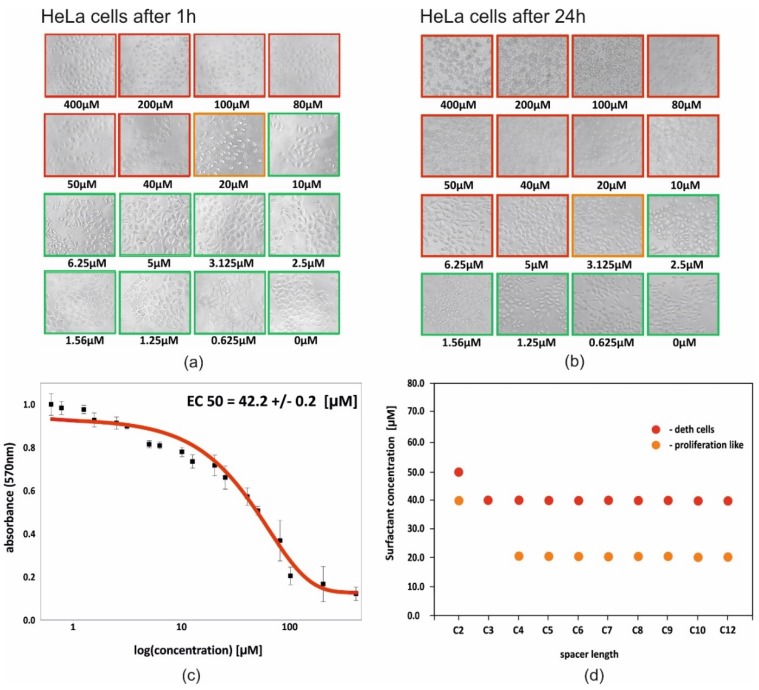
HeLa cells 1 h (**a**) and 24 h (**b**) after addition of C12NC6 solution in the presence of surfactants in increasing concentrations. Red squares correspond to the concentrations that cause cell death, yellow squares correspond to the cell in the proliferation stage. (**c**) MTT test result for Hela cells 1 h after addition of C12NC6 solution. (**d**) Summary of cytotoxicity test results for all tested surfactants. Red spots correspond to surfactant concentrations causing cell death and yellow spots correspond to the critical surfactant concentrations at which the cells are in the proliferation stage.

Toxicity of studied surfactants was tested towards the HeLa model tumor cell line. The results allowed preliminary selection of compounds for transfection (for gene therapy applications). Qualitative assessment of the surfactants was made on the basis of the observed morphological changes in the cells treated with C12NCn surfactants. Microscopic observations made after incubation of cells under given conditions in the presence of C12NCn surfactants revealed significant morphological changes depending on the concentration and structure of surfactants tested (Figure 6d). In the concentration range of surfactants from 0.625 to 10 μM (or to 20 μM for C12NC2), cell survival and morphology were very good. For higher concentrations of surfactants in the medium, in the concentration range from 40 μM (or from 50 μM for C12NC2), cell death was observed. 

The surfactant molecules were absorbed on cell walls and probably caused their perforation, which resulted in cell growth inhibition. For the highest concentrations of surfactants, the breakdown of whole cells was noted. Most surfactants studied (except C12NC3) occurred at concentrations midway between toxic and neutral. The presence of surfactants at an intermediate concentration (low/middle toxic concentration) resulted in small morphological changes in the cells, which assume an oval shape, and caused detachment of cells from the substrate, as observed in the proliferation phase. The surfactant with the shortest spacer group (C12NC2) proved to be the least toxic to the cells tested (Figure 6d), while all other surfactants exhibited higher toxicity. 

For the quantitative assessment of cytotoxicity of the studied gemini surfactants, the colorimetric MTT assay was chosen. This assay gives the effective concentration (EC50), corresponding to the concentration of the tested compound in which proliferation of cells cultured in vitro is inhibited by 50% relative to the control culture. MTT tests revealed that the presence of C12NC6 surfactant in high concentrations in the culture medium had a great influence on the number of living cells. The quantitative and qualitative tests performed for C12NC6 were consistent. EC50 equals 42.2 ± 0.2 μM, which corresponds to 40 µM obtained 1 h after addition of C12NC6 solution (Figure 6a,c).

## 3. Discussion

### 3.1. Conformation of Oligomers in Obtained Stable Lipoplexes

Our gel electrophoresis experiments and circular dichroism studies showed that for the formation of stable C12NCn lipoplexes, a specific p/n ratio is required for both siRNA and dsDNA. The results of the gel electrophoresis experiment indicated better complexing properties of surfactants towards siRNA (for most of them at p/n = 1.5) than dsDNA. A similar effect has been observed previously for the lipoplexes obtained on the basis of imidazolium dichlorides [29,30]. A significant effect of the length of the spacer group of the studied surfactants on the binding efficiency of nucleic acid oligomers was observed. The surfactants with shorter spacer groups (C12NC2–C12NC7) complexed both studied nucleic acids oligomers with similar efficiency (Figure 3). The best complexing properties were found for C12NC4 and C12NC6 surfactants, which may be related to the similar geometrical arrangement of these surfactant molecules in solution [43,44]. The solutions in which stable lipoplexes were formed were usually turbid, as shown also in the photographs taken under an optical microscope (Figure 2).

The circular dichroism experiments showed similar conformational changes in siRNA and dsDNA oligomers induced by the presence of surfactants with shorter spacer groups (C12NC2–C12NC6) (Figure 4a,b). A gradual decrease in the intensity of positive maxima and a shift of the signal towards longer wavelengths in the CD spectra were observed. These changes can be correlated with the compaction of siRNA and dsDNA helices due to electrostatic interaction between the positively charged surfactant heads and the negatively charged surface of oligomers. The CD curves, flattened along the entire course (p/n = 1.5–4), corresponded to the values of the charge ratio at which the electrophoretic mobility stopped and thus stable siRNA and dsDNA complexes were formed. Detailed analysis of the results of the circular dichroism experiment showed that for the lipoplexes formed at p/n = 1.5, the CD spectrum characteristic of A-RNA changed with increasing concentration of a given surfactant. In the presence of four of the selected surfactants (C12NC2, C12NC3, C12NC5, C12NC7) with shorter and odd spacer groups (except C12NC2), the intensity of the positive maximum decreased and the intensity of individual components of this band showed a tendency to equalization (Figure 4c). This effect correlated with the weaker complexation properties of these surfactants (complex for p/n = 2).

The obtained CD spectrum of reference samples of the dsDNA oligomer was typical of the B-DNA form. With increasing concentration of amphiphilic molecules in the solution, the spectrum of circular dichroism changed. Spectral changes, associated with differences in the geometry of surfactants (different length of the linker group), were also observed. For dsDNA lipoplexes based on surfactants with shorter spacer groups (C12NC2–C12NC6), we observed a correlation between the reduction in electrophoretic mobility and the CD signal decrease. We noted gradual flattening of individual bands in the CD spectrum with increasing surfactant concentration. 

A characteristic effect was observed for surfactants C12NC2–C4 at p/n below 2, at which the B-DNA conformation assumes features of the C-DNA form [45]. CD curves showed higher intensity of the minimum at 245 nm relative to the maximum at 282 nm, and the minimum was shifted towards longer wavelengths. This effect is related to the nucleotides transition from BI to BII conformation. In B-DNA, the nucleotides should mostly occur in BI (canonic) conformation, while C-DNA should contain a dominant number of nucleotides in BII conformation. The CD suggest that in B-DNA the nucleotides are in the intermediate state between BI and BII [45,46,47,48]. In the presence of surfactants C12NC5 and C12NC6, no similarities to C-DNA are observed, while the CD signal disappears. 

Very interesting CD spectra were recorded for the dsDNA/C12NC7 system (Figure 4d). For this system, we did not observe a complete flattening of the circular dichroism signal. As the concentration of the surfactant in the solution increased, we observed a gradual reversal of the B-DNA spectrum, which indicates formation of the left-handed form of DNA [35,49]. The circular dichroism spectrum, obtained for dsDNA in mixtures with high concentrations of C12NC7 surfactant (p/n = 1.5–3), was characterized by an extremely high signal intensity. For these lipoplexes characterized by p/n = 3, the inverted spectrum was up to five times more intense than the CD signal obtained for native B-DNA. Becker recorded similar spectra for spermine hydrochloride complexes with calf thymus DNA as a function of NaCl concentration, and defined this conformational form of the oligonucleotide as PSI+. He explained the PSI fast condensation process in the solution as being due to the increased ionic strength [50]. Surfactants, as amphiphilic compounds, have a similar effect on the nucleic acids oligomers in solution. Burchkardt obtained also the same effect for poly-L-histidine with calf thymus DNA complexes [51]. In his work, Burchkardt also referred to Brunner’s results regarding the cholesteric Ψ-DNA form, indicating their similarity [36]. In addition to these pioneering studies on Ψ-DNA forms, this form of oligomer conformation was also observed later [35,49].

Significant differences between the CD spectra, recorded for the systems complexed with the analogous amphiphilic compound, were observed for siRNA and dsDNA lipoplexes obtained using gemini surfactants with longer spacer groups (C12NC8–C12NC12). For the mixtures with siRNA, a decrease in the intensity of the positive maximum at 260 nm was observed, together with spectral shifts towards longer wavelengths and a flattening of the CD signal for the lipoplexes, characterized by the charge ratio p/n corresponding to the effective binding of oligomers.

For the mixtures of dsDNA with surfactants of longer spacers (C12NC8–C12), no flattening of CD curves was observed. However, the signal was shifted towards longer wavelengths and the curves for B-DNA underwent a gradual reverse with increasing concentration of the amphiphilic component in the solution. The CD spectra deformations indicate significant conformation changes and the levorotatory character of the oligomer, which is directly related to deterioration of the complexing abilities. The changes in CD curves were similar to those taking place upon the transition of B-DNA to Z-DNA or X-DNA [35,52,53,54] which has been observed earlier for the mixtures of dsDNA with imidazolium surfactant [30].

### 3.2. Diversity of Structural Ordering in siRNA and dsDNA Oligomer–Surfactant Mixtures

On the basis of the SAXS studies carried out by us for the mixtures of C12NCn surfactants with siRNA oligomer, it was found that the microstructure of these complexes was predominated by micellar or cubic forms. For C12NC2-, C12NC4–C12NC5-, and C12NC8–C12NC10-based systems, broad scattering maxima were observed in SAXS curves, which could be assigned to polydisperse micellar structures formed in the solutions. On the basis of the diffraction maxima, the d parameter for these systems varied in the range 3.72 ≥ d ≤ 3.95 nm. For siRNA lipoplexes based on C12NC3 or C12NC6, a possible cubic phase was identified. However, the number of recorded diffraction peaks was insufficient for unambiguous determination of the symmetry of these structures. 

From our SAXS studies, conducted for the lipoplexes based on dsDNA, in the majority of the studied systems, the lipoplexes occurred in the cubic phase, sometimes coexisting with other phases. For the three surfactants with the shortest spacer groups (C12NC2–C4), we most probably observed the phase Pm3n (Q^223^) [38] with the value of the unit cell parameter in the range a = 7.71–7.76 nm. For the surfactants with longer spacer groups (C12NC5-C10), the Pn3m symmetry structure (Q^224^) [55] with the unit cell parameter a = 7.71–7.90 nm was recognized for most dsDNA lipoplexes. Comparison of unit cells parameters of the cubic phase, determined on the basis of the obtained results, showed that, with increasing of the spacer group length of amphiphilic molecules, the size of the elemental cell was also slightly enlarged (Figure 5). Only for dsDNA/C12NC7 and dsDNA/C12NC12 systems, the presence of inverted hexagonal phase (H_II_) in the solution was observed, which is probably related to the geometry and flexibility of amphiphilic molecules (possible entanglement of longer spacer groups).

The main goal of our study was to develop therapeutic nucleic acid carriers that would have highly efficient transfection capabilities. As demonstrated previously, these abilities depend directly on the morphology and symmetry of the structure of lipoplexes used for this purpose [56]. The fact that most of the lipoplexes, prepared on the basis of the tested mixtures of C12NCn dicationic surfactants with oligomers of siRNA and dsDNA, showed a high degree of organization (cubic or hexagonal structure) is satisfactory. Transfection factors based on lipids, surfactants, and their mixtures are characterized by particularly efficient transfection capabilities [39]. The coexistence of structural phases in the mixtures based on C12NCn surfactants is also promising because of the previously demonstrated improvement in transfection ability for mixtures of similar compounds with DNA (21 bp) in the presence of DOPE [57]. 

**Figure 7:**
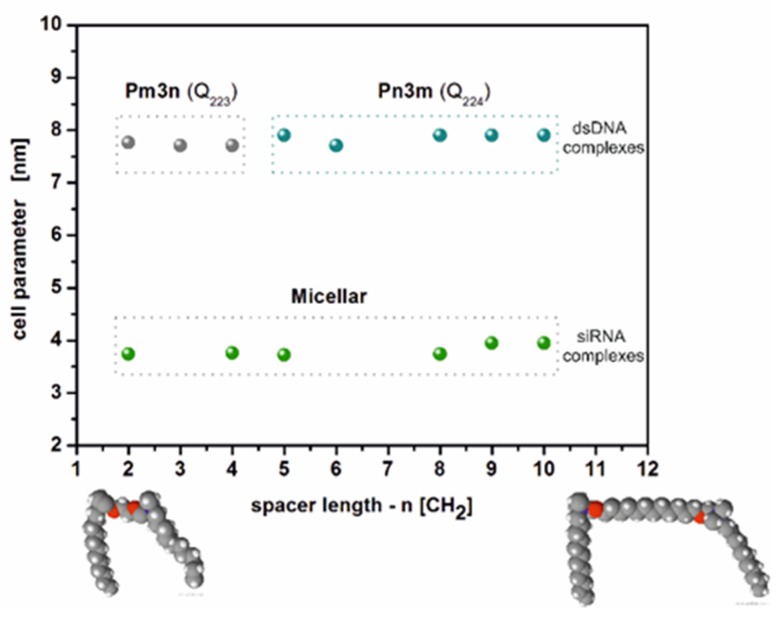
Spacing parameters for the cubic and micellar phase formed in the tested mixtures as a function of surfactants’ spacer length. Molecular models – oxygen (red), nitrogen (blue), carbon (gray) and hydrogen (white).

The SAXS, CD, and OM studies revealed a relationship between the formation of a three-dimensional structure (i.e., formation of cubic phases) and the disappearance of transparency of mixtures in which these phases are present. A typical cubic phase, formed of ammonium-type surfactant molecules in solution, usually should be fully transparent [58]. The presence of nucleic acid oligomers in the solution of some surfactants with long spacer groups may result in a situation when the flexible surfactants molecules are able to form entangled structures and to complex with nucleic acids in a more chaotic manner, generating certain disordered structures. SAXS data, recorded mainly for the systems with p/n ratio from 3 to 4 (C12NC5–C12NC12), were characterized by a relatively low signal-to-noise ratio, which results in flattened diffraction curves (Figure 5d,f,h). Greater turbidity of mixtures based on C12NC2–C12NC4 did not deteriorate the quality of SAXS curves at p/n = 3–4, leading to illegibility of diffraction peaks. On the contrary, their intensity increased (Figure 5b), which may be related to the presence of the cubic structure (Pm3n). Moreover, the surfactants of short spacers (to about 3) are not flexible [59] and show a limited ability to interact with the defected regions of a cubic lattice (Figure 7).

## 4. Materials and Methods 

### 4.1. Synthesis of Gemini Surfactants

**Figure 8:**
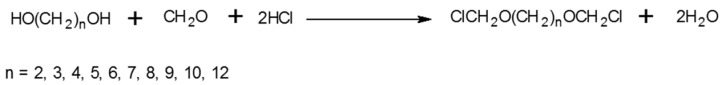
Scheme of α,ω-di(chloromethoxy)alkanes.

The compounds were prepared by dissolving N,N-dimethyldodecylamine in ethyl acetate and adding appropriate amount of α,ω-di(chloromethoxy)alkane. α,ω-Di(chloromethoxy)alkanes (Figure 8) were obtained by passing HCl gas through a mixture of formaldehyde and appropriate α,ω-diol according to the procedure described earlier [60].

**Figure 9:**
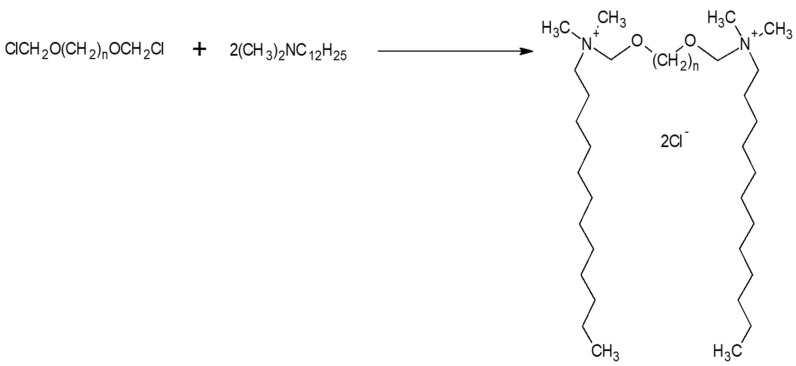
Ammonium gemini surfactants: Synthesized alkanediyl-α,ω-bis[(oxymethyl) dimethyldodecylammonium] chlorides.

All α,ω-di(chloromethoxy)alkanes: 

1,2-di(chloromethoxy)ethane, 1,3-di(chloromethoxy)propane, 1,4-di(chloromethoxy)butane, 1,5-di(chloromethoxy)pentane, 1,6-di(chloromethoxy)hexane, 1,7-di(chloromethoxy)heptane, 1,8-di(chloromethoxy)octane, 1,9-di(chloromethoxy)nonane, 1,10-di(chloromethoxy)decane, and 1,12-di(chloromethoxy)dodecane readily hydrolyzed in the presence of a small amount of water to form HCl, which in turn gives N,N-dimethyldodecylamine hydrochloride. The separation of the quaternization product and hydrochloride is practically impossible. For this reason, all quaternization reactions of N,N-dimethyldodecylamine with α,ω-di(chloromethoxy)alkanes were conducted under strictly anhydrous conditions. The mixture was stirred and heated under reflux for 15 min. After cooling the solution to room temperature, the crude product was separated, extracted with hexane, and the residue dried in vacuum to give the pure product (Figure 9).

### 4.2. Optical Microscopy (OM)

Optical images of complexes were obtained using an Axio Scope A1 Microscope (Zeiss, Germany) in the bright field mode. Portions of 15 µl of solutions were deposited on transparent glass microscope slides. Images were taken under a magnification of 200×.

### 4.3. Agarose Gel Electrophoresis (AGE)

Experiments were performed using Rotiphorese^®^ Unit PROfessional II (Carl Roth, Germany) at 100 V in 2% agarose gel (Carl Roth, Germany), containing ethidium bromide (EtBr) as a fluorescent label. The electrophoretic separation was made for 45 min in 0.5× Tris-borate buffer (45 mM Tris-borate and 1 mM EDTA). All gels were visualized using transmitted UV light and images were taken by a Canon EOS 700D camera. 

### 4.4. Circular Dichroism (CD) Spectroscopy 

Circular dichroism spectra for C12NCn–nucleic acid systems were recorded using a JASCO spectrometer J-815 (Jasco, Japan). These measurements were performed in a standard quartz cuvette (Hellma, Germany) of 0.5 mm path length. Every CD spectrum was an average of five scans in the range 200–350 nm, collected at a scan rate of 100 nm/min.

### 4.5. Small-Angle X-ray Scattering of Synchrotron Radiation (SR-SAXS)

SAXS experiments on DNA and RNA lipoplexes were performed at the P12 BioSAXS beamline of the EMBL Hamburg Outstation (Petra III storage ring at DESY). The detailed experimental parameters are given in Table 2.

All SAXS frames recorded for DNA and RNA lipoplexes were processed (including averaging, normalization, and buffer subtraction procedures) using the program package PRIMUS [61].

### 4.6. Toxicity Assays

For all studied alkanediyl-α,ω-bis[(oxymethyl)dimethyldodecylammonium] chlorides in solution (concentration ranged from 0.625 to 400 µM), the cytotoxicity towards HeLa cells was evaluated. The tested cells were grown according to the standard protocol in DMEM (Dulbecco’s modified Eagle’s medium with L-glutamine, low glucose, Lonza Walkers Ville Inc., USA) supplemented with 10% FBS (fetal bovine serum, EuroClone, Italy) and enriched using appropriate antibiotic/antimycotic agents. We added 1 μL of tested C12NCn surfactant (in the appropriate concentration) to HeLa cells culture (confluence approx. 80%) in 99 μL DMEM medium (without FBS and antibiotics). At the next stage, the cultures, containing C12NCn surfactants, were incubated in an atmosphere of 5% CO_2_ at 37 °C for 1 h and 24 h. Images allowing observation of the morphological changes in the studied cells after incubation with a surfactant were taken under a Zeiss Axiovert microscope (Carl Zeiss, Germany). Quantitative tests on HeLa cells were performed using the MTT Cell Proliferation Assay Kit (Cayman Chemicals, USA). Absorption at a wavelength of 565 nm (MTT assay) was measured using a Tecan Infinite M200pro microplate reader.

## 5. Conclusions

Summing up the obtained results of structural and spectroscopic studies, it should be concluded that the dicationic gemini surfactants—alkanediyl-α,ω-bis[(oxymethyl)dimethyldodecylammonium] chlorides—effectively formed complexes both with siRNA and dsDNA oligomers. However, it was observed that the complexing was somewhat more effective for siRNA duplex. Additionally, the conformation of the siRNA oligomer in lipoplexes was significantly less affected by dicationic surfactants in comparison to dsDNA.

## Figures and Tables

**Table 1:** Analysis of SR-SAXS data and structural phases proposed for dsDNA–C12NCn and siRNA–C12NCn mixtures.

	siRNA				dsDNA			
**Surfactant**	**s [nm^−1^]**	**d or a [nm]**	***hkl***	**Phase**	**s [nm^−1^]**	**d or a [nm]**	***hkl***	**Phase**
**C12NC2**	1.68	3.74		Micellar	1.62 2.01 2.54 3.02	a = 7.76 3.88 3.13 2.47 2.08	200 211 310 321	Pm3n
**C12NC3**	1.62 2.30	a = 7.76 3.88 2.73	200 220	Cubic	1.48 * 1.63 2.01 2.05 * 2.56 2.91 3.04	a = 7.71 4.25 3.85 3.13 3.06 2.45 2.16 2.07	200 211 310 320 321	Pm3n
**C12NC4**	1.67	3.76		Micellar/L	1.63 2.01 2.56 2.91 3.04	a = 7.71 3.85 3.13 2.45 2.16 2.07	200 211 310 320 321	Pm3n
**C12NC5**	1.69	3.72		Micellar	1.59 1.65 * 1.72 * 1.80 * 2.18 2.39 2.67	a = 7.90 3.95 3.81 3.65 3.49 2.88 2.63 2.35	200 220 221 222	Pn3m
**C12NC6**	1.67 2.36	a = 7.52 3.76 2.66	200 220	Cubic	1.63 1.67 * 1.99 2.44	a = 7.71 3.85 3.76 3.16 2.58	200 211 221	Pn3m
**C12NC7**	1.48 1.64	4.25 3.83		Unknown	1.41 2.43 2.76	5.15 2.99 2.63	001 111 002	H_II_
**C12NC8**	1.68	3.74		Micellar	1.59 1.85 2.45 2.77 3.20	a = 7.90 3.95 3.40 2.56 2.27 1.96	200 211 221 222 400	Pn3m
**C12NC9**	1.59	3.95		Micellar	1.59 1.85 2.45 2.77	a = 7.90 3.95 3.40 2.56 2.27	200 211 221 222	Pn3m
**C12NC10**	1.59	3.95		Micellar	1.53 * 1.59 1.82 2.41 2.70	a = 7.90 4.11 3.95 3.45 2.61 2.33	200 211 221 222	Pn3m
**C12NC12**	1.58 1.63	3.98 3.85		Unknown	1.50 2.58 3.02	4.84 2.81 2.40	001 111 002	H_II_

* diffraction peaks not assigned to particular phase

**Table 2:** SR-SAXS experimental parameters.

Detector Type	Pilatus 2M (253 × 288 mm^2^)
Distance to sample	3500 mm
Scattering vector	0.08 > s > 4.5 nm^−1^
Wavelength	λ = 0.154 nm
Number of frames	20

## Abbreviations

AGE	Agarose Gel Electrophoresis
OM	Optical Microscopy
SR-SAXS	Small-Angle X-ray Scattering of Synchrotron Radiation
CD	Circular Dichroism Spectroscopy
dsDNA	Double Stranded Deoxyribonucleic Acid
siRNA	Small Interfering Ribonucleic Acid
DMPK	Myotonic Dystrophy Protein Kinase
FBS	Fetal Bovine Serum
DMEM	Dulbecco’s Modified Eagle’s Medium
